# First observation of a spawning mantle display in a European unionid mussel

**DOI:** 10.1002/ece3.70016

**Published:** 2024-07-15

**Authors:** Sebastian L. Rock

**Affiliations:** ^1^ River Ecology and Management, Department of Environmental and Life Sciences Karlstad University Karlstad Sweden

**Keywords:** endangered species, host attraction, lure, mantle display, Unionida

## Abstract

Freshwater bivalve mussels in the order Unionida are highly endangered ecosystem engineers with a parasitic lifecycle necessitating a fish host to metamorphose from larval glochidia to juvenile mussel. While many species are broadcast spawners and release a large number of glochidia into the water column, many other species have a variety of highly evolved lure mechanisms and mantle displays to attract hosts to ensure a more targeted infestation. Almost all lure mussels are found exclusively in North America, with only one European species (*Unio crassus*) occasionally displaying a host attraction behaviour referred to as *larval spurting*. Here, I present evidence that the depressed river mussel (*Pseudanodonta complanata*) exhibits mantle displays to attract fish to gravid mussels for a targeted infestation, the first description of mantle displays in Europe.

## Graphical abstract

Freshwater bivalve mussels in the order Unionida are highly endangered ecosystem engineers with a parasitic lifecycle necessitating a fish host to metamorphose from larval glochidia to juvenile mussel. Almost all lure mussels are found exclusively in North America, with only one European species (*Unio crassus*) occasionally displaying a host attraction behaviour referred to as larval spurting. Here, I present evidence that the depressed river mussel (*Pseudanodonta complanata*) exhibits mantle displays to attract fish to gravid mussels for a targeted infestation, the first description of mantle displays in Europe.

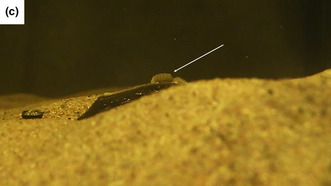

The decline of unionid mussel populations has been largely attributed to factors such as pollution, siltation, habitat loss and general habitat degradation (Lopes‐Lima et al., 2017; Sousa et al., 2023). As obligate parasites on fish, the loss of host fish species has also been raised as contributing factor behind the board population decline of this order as a whole (Modesto et al., 2018). Unionid mussels have a variety of well‐described lure mechanisms to increase infestation rates on host fish which can range from mantle displays to attract fish to gravid mussels, to ornately shaped glochidia coagulates to entice fish to ingest the larva (referred to as *glochidia*; Haag & Warren, 1999; Jones et al., 2023). In Europe, only one mussel species has a documented host attraction behaviour, the endangered thick‐shelled river mussel (*Unio crassus*). Referred to as *larval spurting*, gravid mussels creep into very shallow water by the shore and eject a mixture of water and glochidia towards deeper water, host fish are enticed by this surface movement to then ingest loose glochidia (Aldridge et al., 2023; Vicentini, 2005).

The depressed river mussel (*Pseudanodonta complanata*) is a poorly studied species with a broad distribution covering most of central and northern Europe, and is typically found buried deeply in the sand and silt of slow flowing rivers and lakes (Abraszewska‐Kowalczyk, 2002; Lopes‐Lima et al., 2017; Mcivor & Aldridge, 2007). The species is rare across its entire distribution, with evidence of over 50% population declines in recent decades, and was last listed as Vulnerable in 2011 (Aldridge et al., 2007; Tudorancea, 1972; Van Damme, 2011). The species is particularly susceptible to environmental changes, as it has been reported to have the highest mortality rate of any freshwater mussel following a disturbance event (Ćmiel et al., 2019). Here, I present an ex situ observation of *P. complanata* exhibiting what may be a host attraction behaviour by way of an enlarged, patterned and pulsing excurrent siphon.

Thirty‐one *P. complanata* (size range: 36–67 mm) were collected from the outlet of the river Säveån (57°45′57.3″ N 12°15′12.4″ E) on 25 September 2023, and housed in an aquarium at the Karlstad University aquarium facility as per Rock (2024). On 3 November 2023, six individuals were determined to be gravid and sacrificed for a separate study. On 12 March 2024, three individuals were observed with swollen excurrent siphons (Figure 1a), filled with an off‐white‐coloured material (Figure 1b) and with a visible pattern (Figure 1c). Figure 1d highlights a non‐gravid individual with a less prominent siphon, though still patterned. This off‐white material was collected with a plastic pipette and, when observed under a microscope determined to be a loose coagulate of viable glochidia. Viability of the glochidia was determined by adding a few grains of tale salt (NaCl) to initiate a characteristic snapping stress response. The enlarged siphons were observed pulsating in a manner which did not dislodge the glochidia coagulate (Videos 1, 2, 3). There appeared to be a relationship between the amount of glochidia in the excurrent siphon and the level of siphon activity. Of the three mussels exhibiting mantle displays, the most active had the highest number of glochidia in the siphon (over 250 glochidia per sampling event; Video 1) while the least active had the lowest (<50 glochidia per sampling event; Video 3). One mussel was observed closing the siphon to withhold present glochidia before closing rapidly to reposition and ejecting enough water to displace the surrounding sand, indicating that, if the mussels were broadcast spawners, they could eject the glochidia from the siphon (Video 4). Videos 5 and 6 show non‐gravid *P. complanata* without a mantle display.

**FIGURE 1:**
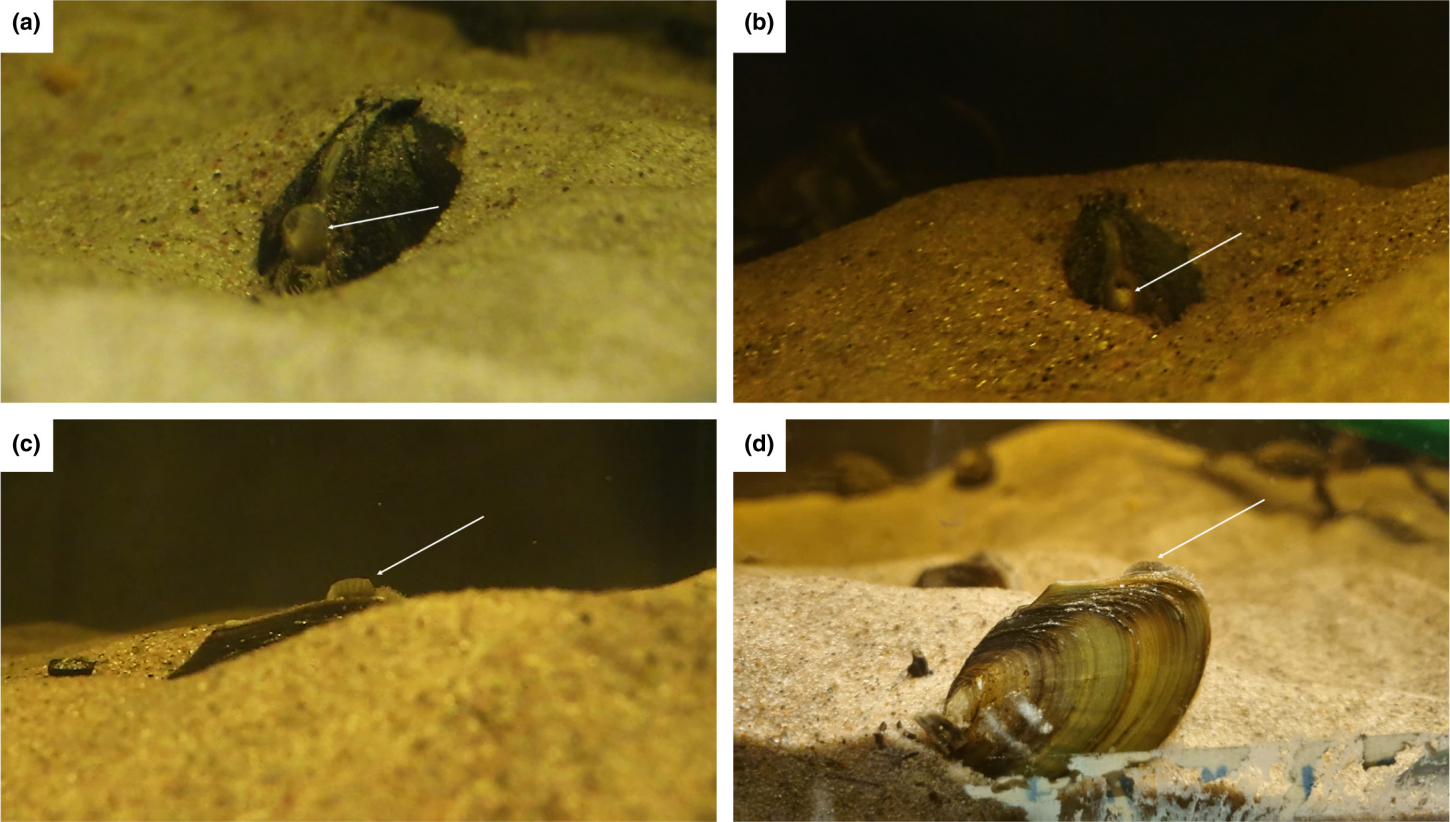
Three quarter‐angle view of a *Pseudanodonta complanata* displaying swollen excurrent siphon with some visible glochidia, indicated with white arrow (a), frontal view of the same mussel with visible glochidia in the excurrent siphon, indicated with white arrow (b), lateral view of a second mussel with visible pattern on the siphon, indicated with white arrow (c) and non‐gravid individual, excurrent siphon indicated with white arrow (d). Images taken by Sebastian L. Rock in the Karlstad University aquarium facility.

**VIDEO 1:** Video of a gravid *Pseudanodonta complanata* with an active mantle display and visible glochidia; at 1:07 in the video glochidia are sampled with a plastic pipette. Video taken by Sebastian L. Rock in the Karlstad University aquarium facility.

**VIDEO 2:** Video of a gravid *Pseudanodonta complanata* with an active mantle display and no visible glochidia. Video taken by Sebastian L. Rock in the Karlstad University aquarium facility.

**VIDEO 3:** Video of a gravid *Pseudanodonta complanata* with an inactive mantle display and no visible glochidia; at 1:02 in the video glochidia are sampled with a plastic pipette. Video taken by Sebastian L. Rock in the Karlstad University aquarium facility.

**VIDEO 4:** Video of a gravid *Pseudanodonta complanata* with an active mantle display and visible glochidia repositioning and not releasing glochidia, observable at 0:45 in the video. Video taken by Sebastian L. Rock in the Karlstad University aquarium facility.

**VIDEO 5:** Video of a non‐gravid *Pseudanodonta complanata* without an active mantle display. Video taken by Sebastian L. Rock in the Karlstad University aquarium facility.

**VIDEO 6:** Video of a non‐gravid *Pseudanodonta complanata* without an active mantle display. Video taken by Sebastian L. Rock in the Karlstad University aquarium facility.

The behaviour observed here is comparable to other mussel species with mantle displays like *Medionidus conradicus* (Lane, 2013) or *Villosa delumbis* (Eads, 2009), and is the first observation of a European mussel species with a mantle display. Patterns were observed on the excurrent siphons of both gravid and non‐gravid mussels, and are therefore not a purely reproductive trait; however, observations of *Epioblasma brevidens* demonstrate that males can also exhibit mantle displays (Jones et al., 2023). It should be noted that *P. complanata* is currently considered to be a broadcast spawner like *Anodonta anatina* and *A. cygnea*, the two most closely related species in Europe. Directed reproductive strategies, like mantle displays, trade low infestation on many fish for a high infestation on fewer fish, and are therefore decrease in efficacy with decreases in host prevalence. Moreover, mantle displays require hosts to visually identify the mussel and then approach it to become infested, which becomes more difficult with decreased water clarity, common in disrupted habitats, further decreasing overall infestation rates (Höök et al., 2020). More research should be conducted on *P. complanata* to validate this observation. An initial host preference survey should be conducted to assess if this species primarily infests benthic or pelagic fish species. Following that, a true experiment should be conducted by exposing potential hosts to gravid and non‐gravid individuals in a series of tests to demonstrate if gravid individuals are approached at a rate higher than non‐gravid individuals.

## AUTHOR CONTRIBUTIONS

**Sebastian L. Rock:** Conceptualization (equal); data curation (equal); methodology (equal); project administration (equal); resources (equal); supervision (equal); validation (equal); visualization (equal); writing – original draft (equal); writing – review and editing (equal).

## FUNDING INFORMATION

This study received funding from the EU LIFE Program (Project acronym: LIFE CONNECTS; LIFE18 NAT/SE/000742) and Karlstad University.

## CONFLICT OF INTEREST STATEMENT

The author declare no conflicts of interest.

## PERMISSION TO REPRODUCE MATERIAL FROM OTHER SOURCES

All pictures in this comment were taken by the author.

## Data Availability

The author has no data to disclose.
